# PLA- and PHA-Biopolyester-Based Electrospun Materials: Development, Legislation, and Food Packaging Applications

**DOI:** 10.3390/molecules29225452

**Published:** 2024-11-19

**Authors:** Cristian Patiño Vidal, Cristina Muñoz-Shugulí, Manon Guivier, Débora Puglia, Francesca Luzi, Adrián Rojas, Eliezer Velásquez, María José Galotto, Carol López-de-Dicastillo

**Affiliations:** 1 Safety and Resources Valorization Research Group (INVAGRO), Faculty of Engineering, Universidad Nacional de Chimborazo (UNACH), Av. Antonio José de Sucre Km 1 1/2, Riobamba 060108, Ecuador; 2 Group for Research and Innovation in Food Packaging, Riobamba 060107, Ecuador; cristina.munoz@espoch.edu.ec; 3 Faculty of Sciences, Escuela Superior Politécnica de Chimborazo (ESPOCH), Panamericana Sur km 1 1/2, Riobamba 060106, Ecuador; 4 Polymer Chemistry and Materials, Adolphe Merkle Institute, University of Fribourg, 1700 Fribourg, Switzerland; manon.guivier@unifr.ch; 5 Materials Science and Technology Laboratory, Civil and Environmental Engineering Department, University of Perugia (UNIPG), 05100 Terni, Italy; debora.puglia@unipg.it; 6 Department of Science and Engineering of Matter, Environment and Urban Planning (SIMAU), Polytechnic University of Marche (UNIVPM), 60131 Ancona, Italy; f.luzi@staff.univpm.it; 7 Packaging Innovation Center (LABEN), University of Santiago of Chile (USACH), Santiago 9170201, Chile; adrian.rojass@usach.cl (A.R.); eliezer.velasquez@usach.cl (E.V.); maria.galotto@usach.cl (M.J.G.); 8 Center for the Development of Nanoscience and Nanotechnology (CEDENNA), University of Santiago of Chile (USACH), Santiago 9170201, Chile; 9 Packaging Laboratory, Institute of Agrochemistry and Food Technology (IATA-CSIC), 46980 Valencia, Spain

**Keywords:** electrospinning, polylactic acid, polyhydroxyalkanoates, compostability

## Abstract

The high accumulation of plastic waste in the environment has led to great interest in biodegradable polymers, such as polylactic acid (PLA) or polyhydroxyalkanoates (PHAs). Their benefits, combined with the application of electrospinning technology, represent an innovative proposal for the food packaging industry. This article provides a comprehensive review of the latest developments of PLA- and PHA-biopolyester-based electrospun materials for food packaging applications, summarizing the reported technologies, material properties, applications, and invention patents. In addition, the legislation used to assess their biodegradability is also detailed. Electrospun packaging materials are largely developed through uniaxial, coaxial, emulsion, multiaxial, and needleless techniques. PLA- and PHA-biopolyester-based electrospun materials can be obtained as single and multilayer packaging structures, and the incorporation of natural extracts, organic compounds, and nanoparticles has become a great strategy for designing active food packaging systems. The biodegradability of electrospun materials has mainly been evaluated in soil, compost, and aquatic systems through ASTM and ISO normatives. In this review, the dependence of the biodegradation process on the polymer type, conditions, and test methods is clearly reviewed. Moreover, these biodegradable electrospun materials have shown excellent antioxidant and antimicrobial properties, resulting in a great method for extending the shelf life of fruits, bread, fish, and meat products.

## 1. Introduction

In the last century, the plastics obtained from non-renewable feedstock have become a key tool in the packaging industry. The production of plastics in the world was approx. 391 million tons in 2021, and this will increase by 1.5 times by 2050 [1]. The high use of plastics has been associated with their valuable properties, highlighting their low cost, durability, mechanical and chemical resistance, low permeability to water and gases, transparency, high performance, and light weight [1]. These characteristics have made plastic packages highly essential to ensure the safety and quality of foods. The traditional processing techniques used to produce plastic packages include extrusion, film blowing, injection molding, and coating [2]. However, the extensive degradation times of plastic packages have caused high waste plastic accumulation in the ecosystem, and this fact has mainly arisen because strict management regulations have been not implemented [3]. Wu et al. (2021) reported that the food packaging industry generated the highest amount of plastic waste during 2018 compared to the waste produced by the textile, transportation, building, and construction sectors, and this was largely derived from products with a short lifetime (up to 6 months) [4]. To address this drawback, transitioning towards a sustainable development model by 2030 based on the Circular Economy proposed by the European Union and the development of eco-friendly food packaging materials based on polylactic acid (PLA) or polyhydroxyalkanoates (PHAs) have been the most used alternatives to plastic waste management [5,6,7]. Although PLA- and PHA-biopolyester-based packaging materials exhibit deficiencies as food packaging due to their low physical–chemical and barrier properties, lately, they have become one of the most explored options, as the conventional recycling of food packages is limited because great parts of packages are complex multilayer structures difficult to separate into components and reprocess [1,8].

PLA is a linear aliphatic thermoplastic polymer widely used in the food packaging industry. Its advantageous characteristics of biodegradability, compostability, and safety and properties similar to conventional polymers, such as poly (ethylene terephthalate) (PET), have allowed it to be considered as the main biodegradable alternative in the food packaging industry [8,9]. On the other hand, PHAs constitute a wide range of linear aliphatic polyesters. PHAs can be obtained naturally, biochemically, or chemically, and they also exhibit attractive properties, highlighting their biodegradability, compostability, transparency, and safety [1,10]. PLA and PHAs have been transformed into food package prototypes through traditional melt-processing-based techniques, and their biodegradation has been tested in fresh water, marine water, soil, and compost [11,12,13].

On the other hand, in the last decade, electrospinning has emerged as a cutting-edge technique able to produce biodegradable and compostable packaging materials. Electrospinning is a versatile, efficient, cost-effective, and scalable technology able to produce nanofiber- and microfiber-based films with a high surface-to-volume ratio, adjustable surface characteristics, and adequate physical–mechanical and barrier properties for food packaging [14]. Electrospinning can also protect thermolabile compounds or incorporate reinforcements into the polymeric matrix, as they are encapsulated into fibers. In addition, reinforced or functionalized electrospun materials can be added into multilayer systems to obtain biodegradable packaging materials with enhanced properties [15,16,17].

This article refers to the latest developments of PLA- and PHA-biopolyester-based food packaging materials obtained through electrospinning. The aim of the study is to thoroughly review the electrospinning systems used to obtain these packaging materials and understand their main physical and mechanical properties, the legislation and standards used to assess their biodegradability, and the tests to confirm their functionalities through in vitro and in vivo conditions. This review also reports the technologies available in the food packaging plastic market or to be transferred to the industry, described as invention patents associated with these electrospun materials.

## 2. Electrospinning and Nanofibers

### 2.1. Definition, Characteristics, and Influencing Factors

Electrospinning is a simple, versatile, and efficient technique used to produce nano-and microfibers with a high surface-to-volume ratio and porosity. The process involves the application of an electric field between a needle tip connected to a polymeric solution by a syringe and a collector that elongates the drop into a conical shape, resulting in thin fibers that solidify on the oppositely charged collector in the form of a mesh material [18,19].

Electrospinning can be applied in various fields, including filtration, tissue engineering, drug delivery, and energy storage [19]. In the last decade, the development of electrospun materials for food packaging purposes has gained much interest. This fact is due to their several advantages when compared to other techniques that produce fibers and/or process polymers. For instance, the electrospinning process is able to produce ultrafine fibers with excellent control over fiber morphology. Thus, their high surface-area-to-volume ratios are highly advantageous in several applications, such as for the controlled release of bioactive agents or as adsorbents in other applications [20,21], while controlling the fiber morphology allows for the handling of specific properties thanks to their homogeneity and uniform fiber distribution, such as enhanced mechanical strength and barrier properties. By controlling the composition and structure of these fibers, it is possible to create packaging materials that provide better protection against oxygen, moisture, and other environmental factors, thus extending the shelf life of packaged food products. Electrospun fibers can be designed with a controlled porosity to regulate gas and moisture permeability. This tunability enables the development of packaging materials that maintain the optimal gas composition and humidity levels within a package, preserving the freshness and quality of foods [22].

Electrospinning also presents a broad versatility in polymers, including the processing of synthetic and natural polymers dissolved in either organic or aqueous solutions, and it is a technology with potential applications in several fields, including pharmaceuticals, biomedical products, and food packaging [23,24]. Unlike traditional polymer processing techniques, which involve the use of high temperatures for melting-based processes, electrospinning allows for processing thermolabile biopolymers based on protein and carbohydrates, which cannot be treated by conventional processes [14,25]. Several studies have developed electrospun fibers with plant proteins, such as gluten, zein, soy, and amaranth proteins, and many of them have used this technique as a method for the encapsulation of active compounds to develop active packaging systems. Thus, electrospun fibers can be loaded with active agents, such as antioxidants and/or antimicrobial compounds, to actively preserve the quality and safety of packaged food products [9,26,27].

Active electrospun fibers offer a wide variety of advantages, highlighting the protection of active compounds sensitive to polymer processing conditions (high temperatures) or environmental conditions (light, oxygen, etc.) and the formation of homogeneous fibers with adequate dispersion of the active agent, with high surface-area-to-volume ratios and a certain controlled release [28]. Despite this, their disadvantages include industrial scale-up production, which is currently possible but scarce due to the high energy consumption, solvent selection, and low production rates of this process. Nevertheless, the interest in scaling up this technology is driving advances in strategies to increase jet production and productivity [23]. Several companies already offer commercial products based on electrospun materials, such as the protein electrospun nanofibers offered by Holmarc Opto-Mechatronics (Kerala, India) or the Spanish Company Nadetech Innovations (Navarra, Spain), able to produce automatized systems with controllability and a high accuracy and reproducibility.

The characteristics of the resulting fibers, such as their morphology, diameter, alignment, and properties, are dependent on several factors, including both the properties of the polymeric solution and the parameters of the electrospinning process. The type of the polymer, its concentration, its molecular weight, the solvent, the presence of additives, and their rheological properties, such as viscosity, conductivity, and surface tension, are some of the main factors to be considered [29]. Higher-molecular-weight polymers typically yield fibers with a greater mechanical strength, while variations in concentration and viscosity affect the fiber diameter and morphology. The choice of solvent or solvent mixture used to dissolve the polymer also affects the electrospinning process. Solvent properties such as volatility, polarity, dielectric constant, and evaporation rate influence the electrospinnability of the polymer solution, as well as the rate of solvent evaporation during fiber formation [30]. Moreover, the addition of additives, such as surfactants, cross-linking agents, or functional nanoparticles, can modify the properties of the electrospinning solution and influence the electrospinning process. Functionalization strategies can impart specific functionalities to the electrospun fibers, enhancing their performance for targeted applications.

On the other hand, the processing parameters to be optimized during the electrospinning process are the flow rate, the applied voltage, the distance between the collector and the needle, the needle type and gauge, and the type of collector, in addition to the environmental conditions (temperature and humidity) [31]. These parameters affect the stretching and elongation of the polymer jet, as well as the evaporation of the solvent, ultimately influencing the fiber diameter, alignment, morphology, and uniformity. The needle geometry, including its shape, size, and number of orifices, and the collector configuration impact the electrospinning process. The configuration and properties of the collector influence the deposition and alignment of the electrospun fibers. Collectors can be stationary or rotating, conductive or non-conductive, flat or cylindrical, and coated or uncoated, affecting the arrangement, density, and orientation of the collected fibers [32,33]. Finally, optimal environmental conditions help to maintain stable electrospinning parameters and facilitate solvent evaporation, contributing to the formation of high-quality fibers.

### 2.2. Types of Electrospinning Systems Used for the Development of Packaging Materials

The electrospinning technique for biodegradable materials has allowed for the production of conventional and functional materials for use in food packaging [14]. The type of spinning solution or needle defines the operation technique, and thus, the structure of the fibers and properties of the material. The main electrospinning modifications are described as follows.

**Figure 1:**
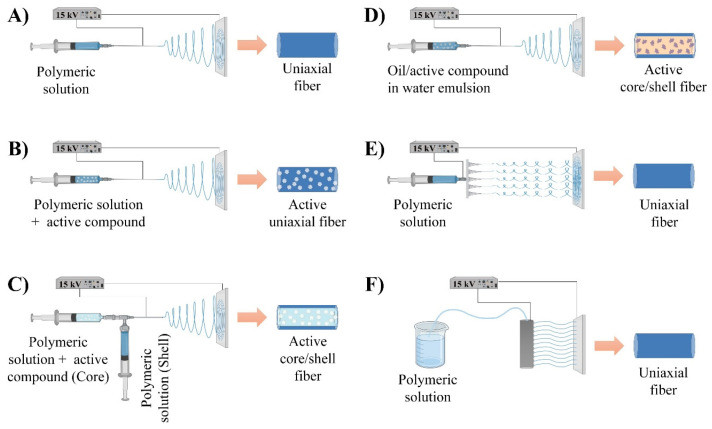
Electrospinning processing based on systems: (**A**) uniaxial, (**B**) uniaxial to encapsulate active compounds, (**C**) coaxial, (**D**) emulsion, (**E**) multiaxial, and (**F**) needleless.

Figure 1A shows the most traditional system, also known as “uniaxial”, which uses a single needle to electrospun a homogeneous polymeric solution and obtain fibers [34]. This technology allows for obtaining eco-friendly mats from biodegradable polymeric solutions. Furthermore, the main usefulness of this technique is the easy and effective encapsulation of active compounds into fibers, as shown in Figure 1B [26]. Thus, nanofibers with a sustainable release of compounds have been developed through the direct incorporation of simple (active compounds) or complex (inclusion complexes) substances into the electrospun structure [26,33]. However, some developments have shown a reduced compatibility between the active compound and the polymer or sensitivity to environmental conditions, favoring a burst release at early stages [35,36]. A novel strategy used to address this drawback is a slight modification in the needle configuration to give rise to coaxial electrospinning.

As depicted in Figure 1C, coaxial electrospinning involves the use of two concentric needles and two polymeric solutions to obtain fibers with a core–shell structure. This complex structure comprises a polymeric core layer loaded with the active compound and a polymeric shell layer that acts as a physical and protection barrier for the active compound. Thus, the gradual diffusion of the active compound through the polymeric matrix promotes its sustained long-term release [33,34]. This highlights that coaxial electrospinning also allows for electrospun non-polymeric substances/compounds (essential oils, natural extracts, and vitamins) without spinning properties and polymers without the ability to be spun [29]. Some studies have applied coaxial electrospinning to produce biodegradable mats for food packaging [11,37,38]. Nevertheless, the industrial scale of this system is restricted by its quite intricate configuration, since attaining alignment for the jet and the consistent spinning of the solution to obtain the material are strong challenges [34].

Electrospinning systems based on emulsion, multijet, and needleless techniques have also been used to produce biodegradable mats. However, their usefulness is minimal compared to uniaxial and coaxial electrospinning. PLA has been the most processed polymer through these technologies [32,39,40]. This fact is related to the potential applications of these configurations in the biomedicine and cosmetic areas, where biocompatible polymers are largely used. Emulsion electrospinning is based on the one-nozzle direct spinning of stable emulsions (oil-in-water or water-in-oil) to obtain core–shell fibers (Figure 1D). In this system, the selection of appropriate polymers for emulsion formation, a low productivity, and processing difficulties due to the characteristics of the polymeric emulsions are the main limiting factors for its industrial application [25,33]. On the other hand, Figure 1E shows the configuration of multijet electrospinning. This system comprises the integration of several nozzles to faster inject the polymeric solution. The primary goal is to enhance the nanofiber manufacturing efficiency [34]. However, the presence of many nozzles also promotes repulsion and interference among adjacent jets, thus leading to a lack of evenness and degradation of the fibers. To address this drawback, needleless electrospinning has recently arisen as an alternative. Rotating spinnerets have been the most common way to implement this technique. Needleless electrospinning consists of the active agitation of the spinning solution while a high voltage is applied. This fact allows for the formation of several Taylor cones that give rise to polymer jets from their tips, who are attracted to the collector and finally convert into fibers (Figure 1F) [25,34].

## 3. Biodegradable PLA- and PHA-Based Electrospun Materials

PLA is a rigid thermoplastic widely used in the food packaging industry due to its biodegradability, sustainability, compostability, safety, and biocompatibility. It is a linear aliphatic thermoplastic polymer obtained from the polymerization of L- and D-lactic acid isomers that are produced by the fermentation of starch coming from sugarcane, potatoes, and corn [8]. Due to PLA exhibiting properties similar to PET or polystyrene, its application in the plastic industry has been widely researched [9]. On the other hand, polyhydroxyalkanoates are a group of linear aliphatic polyesters produced from the fermentation of sugar and lipids. PHAs are composed of monomeric units of R-hydroxyalkanoic acids. The most important PHAs are as follows: poly(3-hydroxybutyrate) (PHB), poly(3-hydroxybutyrate-co-3-hydroxyvalerate) (PHBV), poly(3-hydroxybutyrate-co-3-hydroxyhexanoate) (PHBHHx), poly(3-hydroxybutyrate-co-4-hydroxybutyrate) (P34HB), and poly(3-hydroxybutyrate-co-3-hydroxyvalerate-co-3-hydroxyhexanoate) (PHBVHHx) [10]. PHAs are obtained from different bacteria, and they can be readily degraded in different environments (fresh water, marine water, soil, and compost). PHAs also show attractive properties such as non-toxicity, biodegradability, compostability, biocompatibility, transparency, toughness, and elasticity [10,41]. Both PLA and PHAs have been processed through electrospinning for food packaging purposes [11,12,21,42].

PLA- and PHA-biopolyester-based materials are highly susceptible to degradation (loss of structure) by biological activity, resulting in smaller matrixes (micro- and nanoplastics) [43]. Thus, the biodegradability of these electrospun materials can be defined as their ability to be degraded by living organisms. The biodegradation of an electrospun material involves an enzymatic hydrolysis process catalyzed by microbial secretases that gives rise to the breakdown of the matrix into water (H_2_O), carbon dioxide (CO_2_), and biomass [3]. Taking into account the plastic biodegradation process described by Wang et al. (2024), an electrospun mat can be biodegraded in the following ways: (i) the formation of film microorganisms on the mat, (ii) the macroscopic disintegration of the material into smaller particles by action secretases, (iii) the depolymerization of the matrix by the scission of linkages between the polymer chains, and (iv) the assimilation and mineralization of small molecules for conversion into biomass [43]. A relevant characteristic of the end products resulting from complete biodegradation is their non-ecological toxicity risk. Biodegradation occurs in a timescale short enough (maximum 6 months) that it does not lead to lasting harm or waste accumulation in the environment [3,43].

Depending on the application of PLA- and PHA-biopolyester-based electrospun materials, they have three possible resulting end-of-life scenarios. The first would be in-soil degradation, where the mat used as agricultural soil-biodegradable plastic mulch films can be treated. The second disposition is industrial composting. In this process, electrospun material waste coming from packaging materials can be treated under highly controlled conditions of temperature and humidity. The third would be by anaerobic digestion, where electrospun waste can be anaerobically biodegraded [44].

## 4. Designing PLA- and PHA-Biopolyester-Based Electrospun Materials

### 4.1. Single Electrospun Materials

As Table 1 shows, single mats composed of uniaxial electrospun nanofibers have been the most developed materials in recent years. Their concentrations of polymeric solutions have been at nearly 10% (*w*/*v*). Also, they have been prepared from single polymers (PLA and PHBV) or blends (PHBV/PBAT and PLA/PHB), as well as from single or mixtures of solvents, such as dimethylformamide (DMF), dichloromethane (DCM), chloroform (CLF), and 2,2,2-trifluoroethanol (TFE) [45,46]. The addition of natural extracts, nanoparticles, and inorganic and organic fillers has also been implemented as a strategy to afford functionality to these materials. Regarding processing conditions, flow rates from 0.3 to 6 mL/h, distances between 12 and 20 cm, and voltages from 9 to 20 kV have allowed for obtaining ultrathin (200 nm) and thicker (2.5 µm) nanofibers [47,48]. Thus, these conditions have been combined to produce electrospun materials with excellent physical–chemical, thermal, surface, and mechanical properties.

### 4.2. Multilayer Electrospun Structures

The development of multilayer structures has been a way to enhance the physical–chemical and barrier properties of biodegradable materials. Strengthened properties have arisen from the higher tortuosity of materials with the presence of different layers and/or the addition of particles [16,49].

In recent years, multilayer biodegradable materials containing electrospun layers have been minimally developed. Table 1 displays various research works based on the development and characterization of different types of electrospun fibers obtained by using PLA and PHA biopolymers. The table also includes the conditions used during the electrospinning process, the type of properties that were evaluated, and the principal results.

**Table 1:** Types of biodegradable electrospun materials.

Single Electrospun Materials					
Structure	Electrospun Polymer	Conditions	Evaluated Properties	Main Findings	Reference
Uniaxial	PLA 8% (*w*/*v*)	* DCM:DMF (3:1) ** HNTs-Cu^+2^ *** 0.6 mL/h, 12 cm, 17 kV	SEM, TEM, FTIR, XRD, DSC, WCA, Mechanical (σ_M_, ε_B_, E)	Continuous nanofibers (350–690 nm) with good thermal stability and improved mechanical and hydrophobic properties	[45]
Uniaxial	PHBV 12% (*w*/*v*)	* CLF *** 0.5–5 mL/h, 16 cm, 9–20 kV	SEM, FTIR, WCA, Mechanical (σ_M_, ε_B_)	Thicker nanofibers (2.5 µm) without beads produced under conditions of 20 kV, 0.5 mL/h, and 16 cm	[47]
Uniaxial	PLA 15% (*w*/*v*)	* DCM:DMF (2:1) ** Ag_2_O-hemp fibers *** 3 mL/h, 15 cm, 14 kV, roller speed 40 rad/s	SEM, FTIR, XRD, TGA, WCA, WVTR, Mechanical (σ_M_, ε_B_, E)	Uniform nanofibers (770–880 nm) with improved mechanical properties, low hydrophobicity, and high water vapor permeability	[50]
Uniaxial	PHBV 10% (*w*/*v*)	* DCM:DMF (2:1) ** TiO_2__humic substances NPs *** 3 mL/h, 20 cm, 18 kV	SEM, TEM, XRD, TGA, WCA	Smooth composite fibers (0.9 µm) with low hydrophobicity, high crystallinity, and thermal stability	[51]
Uniaxial	PLA Not reported	* DCM:DMF (2:1) ** *Capparis spinosa* L. extract *** 0.8 mL/h, 13 cm, 15 kV	SEM, FTIR, WCA, Mechanical (σ_M_, ε_B_)	Uniform nanofibers (200–377 nm) with low hydrophobicity and high flexibility	[48]
Uniaxial	PHBV/PBAT 13% (*w*/*v*)	* CLF:DMF (7:3) ** TiO_2_. *** 1.5 mL/h, 15 cm, 17 kV	SEM, FTIR, DSC, WCA, Mechanical (σ_M_, ε_B_, E)	Uniform nanofibers (360–500 nm) with improved mechanical, hydrophobicity, and thermal properties	[52]
Uniaxial	PLA 15% (*w*/*v*)	* DMF:DCM (3:7) ** Perilla essential oil *** 0.3 mL/h, 20 kV	SEM, FTIR, DSC, TGA, WCA, WVP, OP, Mechanical (σ_M_, ε_B_, E)	Uniform nanofibers (312–396 nm) with improved thermal, mechanical, water vapor permeability, and hydrophobicity properties	[53]
Uniaxial	PLA 10% (*w*/*v*)	* DCM:DMF (7:3) ** TiO_2_. *** 0.5 mL/h, 18–20 cm, 18 kV	SEM, FTIR, DSC, WCA, ΔE, opacity, Mechanical (σ_M_, ε_B_)	Rough fibers (0.1–1 µm) with high crystallinity, opacity, and improved mechanical properties	[54]
Coaxial	PLA/PHB 8% (*w*/*v*)	* CLF:DMF (8:2) ** OLA *** 1 mL/h, 14 cm, 11 kV	SEM, XRD, DSC, TGA, Mechanical (σ_M_, ε_B_, E)	Uniform nanofibers (222–440 nm) with improved thermal properties and high crystallinity	[12]
Uniaxial	PHBV 10% (*w*/*v*)	* TFE ** Cyclodextrin inclusion complexes *** 6 mL/h, 20 cm, 18 kV	SEM, TGA, transparency, Mechanical (σ_M_, ε_B_, E)	Beaded nanofibers (0.87–1.22 µm) with good thermal properties	[46]
**Multilayer Electrospun Materials**					
**Structure**	**Electrospun** **Polymer**	**Conditions**	**Evaluated** **Properties**	**Main Findings**	**Reference**
Trilayer Extruded PLA Electrospun PLA/PLA Coating Chitosan	PLA 10% (*w*/*v*)	* CLF:DMF (7:3) ** LAE *** 0.75–1.8 mL/h, 14 cm, 18–20 kV	FESEM, WCA	515 µm thick and low hydrophobicity	[13]
Trilayer Extruded PLA Electrospun PLA/PLA Coating-Chitosan	PLA 10% (*w*/*v*)	* CLF:DMF (7:3) ** LAE *** 0.75–1.8 mL/h, 14 cm, 18–20 kV	ΔE, Opacity, FTIR, DSC, WCA	Highly opaque and white material with a thickness of 515 µm and increased crystallinity	[49]
Quadrilayer Extruded PHBV or PHB blend Electrospun PHBV CNC coating Extruded-PHBV	PHBV 8% (*w*/*v*)	* CLF:Butanol (75:25) ** OEO + ZnO nanoparticles *** 45 mL/h, 30 cm, 22 kV	SEM, ΔE, Opacity, WVP, OP, Mechanical (σ_M_, ε_B_, E)	150 µm thick, good transparency, low water vapor and oxygen permeability, and improved mechanical properties	[55]
Trilayer Electrospun PHBV CNC coating Electrospun PHBV	PHBV 10% (*w*/*v*)	* CLF:Butanol (75:25) ** OEO + ZnO nanoparticles *** 6 mL/h, 20 cm, 20 kV	SEM, WVP, OP Mechanical (σ_M_, ε_B_, E)	100 µm thick, low oxygen permeability, and improved mechanical properties	[15]
Trilayer Extruded PHB Electrospun PHBV Extruded PHBV	PHBV 10% (*w*/*v*)	* TFE ** eugenol *** 6 mL/h, 15 cm, 15 kV	SEM, TGA, WCA, WVP	70 µm thick, high hydrophobicity, mechanical resistance, and low water vapor permeability	[16]
Quadrilayer Extruded YPACK210 Electrospun PHBV CNC coating Extruded YPACK210	PHBV 8% (*w*/*v*)	* CLF:Butanol (75:25) ** OEO + ZnO nanoparticles *** 6 mL/h, 25 cm, 18.5 kV	SEM, ΔE, Opacity, WVP, Mechanical (σ_M_, ε_B_, E)	150 µm thick, high transparency and water vapor barrier, and moderate mechanical properties	[17]
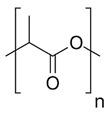 PLA		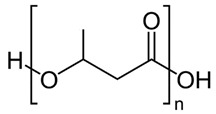 PHB		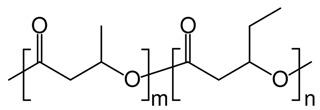 PHBV	

* Solvents. ** Additive. *** Parameters. HNTs-Cu^+2^: natural halloysite nanotubes loaded with Cu^+2^. OEO: oregano essential oil. OLA: oligomeric lactic acid. LAE: ethyl lauroyl arginate. DMF: dimethylformamide. DCM: dichloromethane. CLF: chloroform. TFE: 2,2,2-Trifluoroethanol. σ_M_: tensile strength. ε_B_: elongation at break. E: Young’s modulus. The chemical structures of PLA and PHAs (PHB and PHBV) used to obtain the electrospun materials are shown in the last row.

Trilayer structures with an intermediate electrospun layer loaded with organic substances, nanoparticles, or essential oils have been the most used. Additives have been added to improve the physical–mechanical performance of full materials and promote their functionality. Active electrospun layers have been mainly prepared into blend solvents with polymeric concentrations at 10% (*w*/*v*). Furthermore, high flow rates, large distances, and high voltages have been applied to produce active electrospun layers. On the other hand, external layers have consisted of extruded films or coatings. Finally, multilayer structures have been compacted by hot pressing [15], lamination [55], or coating [13] with a thickness between 70 and 515 µm.

## 5. Characteristics of PLA- and PHA-Biopolyester-Based Electrospun Materials

The morphology and surface properties of electrospun biodegradable materials have been widely evaluated. Scanning Electronic Microscopy (SEM) and Transmission Electronic Microscopy (TEM) have been the most used techniques to observe the morphology, size, structure, and surface of nanofibers and multilayer materials. As Table 1 shows, continuous and beaded nanofibers with different surfaces (smooth or rough) and diameters (thinner and thicker) have mainly been obtained [45,51]. SEM has also allowed for verifying the effective compaction of multilayer materials. Smooth and compact surfaces have been produced by lamination or hot pressing [15,16], while heterogenous surfaces have been been produced through coating [13]. On the other hand, the surface structure influences the surface properties of electrospun materials, and this effect has been evaluated by water contact angle measurements (WCAs). High WCA values (>65°) generally show the hydrophilic performance of a material [13]. Single and multilayer electrospun materials have shown hydrophilic and hydrophobic behaviors, and this performance is mainly influenced by the presence of active substances or fillers (Table 1).

Thermal stability and the presence of thermal transitions during the heating of electrospun materials have been assessed through thermogravimetric analysis (TGA) and differential scanning calorimetry (DSC) (Table 1). The high thermal stability of nanoparticles or organic compound–polymer interactions has promoted a higher thermal stability and crystallinity of polymeric blends of PHBV/PBAT and PLA [52,54]. The same effect was observed with the incorporation of Perilla essential oil into PLA [53]. To explain these thermal phenomena, Fourier transform infrared spectroscopy (FTIR) and X-ray diffraction analyses (XRD) have been conducted. The chemical interactions between polymeric matrixes and additives, as evidenced by FTIR analysis, have confirmed the better thermal stability of materials. Instead, the crystallinity of electrospun materials obtained from DSC analysis has been also validated through XRD analysis. Table 1 details some electrospun materials where these phenomena have been evidenced.

The mechanical performance of electrospun materials has been evaluated by tensile tests, and the results are typically reported as tensile strength (σ_M_), elongation at break (ε_B_), and Young’s modulus (E). Table 1 shows that single electrospun materials have been widely tested, with all studies showing an improvement in their mechanical properties when active compounds and fillers were added in such polymeric matrixes. Likewise, this improvement effect has been evidenced in FTIR, XRD, DSC, and TGA analyses where the chemical polymer–additive interactions favored a higher ordering of polymeric chains and/or reinforcement of material [45,50].

The optical and barrier properties of electrospun materials have been also evaluated. Single electrospun mats have shown a high opacity and white color. In contrast, multilayer structures have produced different results. Coated materials have shown a high opacity and white color [13]. However, laminated or hot-pressed materials have exhibited a good transparency and minimal color changes (ΔE) in comparison with coated materials [17,55]. Regarding barrier properties, water vapor permeability (WVP) and oxygen permeability (OP) have been mainly tested for multilayer structures. The influence of relative humidity on the barrier properties of electrospun materials has been the main factor studied. Laminated or hot-pressed structures have been observed as the materials with better barrier properties. This effect is the result of the presence of different layers, which creates a higher tortuous pathway to the passing of permeants through the polymeric matrix [15,55].

## 6. Legislation on PLA- and PHA-Biopolyester-Based Electrospun Materials

### 6.1. Normative and Laws

The assessment of the biodegradability characteristics of biopolymeric materials is rather challenging, as it depends on many factors, often resulting in disagreements between results from lab and field testing. The complex nature of electrospun materials importantly compromises test results due to their variable porosity, water solubility and WVP, cross-linking level (chemically, photochemically, or thermally obtained), and processing techniques (lamination, heat sealing, and blending polymers) [56,57]. A high surface-area-to-volume ratio, nanoporous structure, high porosity, and high absorption capacity make electrospun materials more sensitive to surrounding changes in acidity/alkalinity; thus, it is possible to better control the advance of biodegradation in such systems [58]. Furthermore, one of the main challenges in electrospinning is selecting safe and eco-friendly End of Life (EoL) options, so it is crucial to consider the biodegradation of such materials in different environments [33].

A main benefit of biodegradable polymers compared to conventional ones is their capability to undergo biodegradation by microbial activity and environmental factors. However, biodegradation is dependent on the polymer type, conditions, and test methods. Appropriate standards are needed to validate biodegradability and compostability claims for bioplastics [59]. Indeed, the methodologies currently used to assess biodegradability are organized according to the place where the plastics are being disposed (soil, compost, and aquatic systems), without giving any specific details or information on how to deal with the electrospun membranes [60].

**Figure 2:**
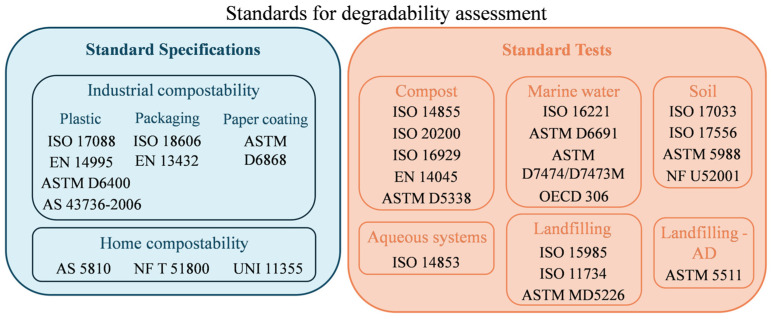
Standard and research methods for the assessment of bioplastics’ degradability adapted from Folino et al. (2023) [61] used under a Creative Commons CC--BY license.

International standards have been established to systematically evaluate the biodegradation and compostability of plastic materials (Figure 2). Recently, it was determined that a combination of standards should be used to substantiate and certify compostability claims in different environments [60,61]. For packaging purposes, at the European level, the EN 13432 standard [62] establishes the requirements for packaging recoverable through composting and biodegradation. The requirements are at least 90% disintegration after 12 weeks, 90% biodegradation (CO_2_ evolvement) in 6 months, and the inclusion of tests on ecotoxicity and heavy metal content.

In all cases, it is acceptable for test specimens to be in the form of films, pieces, fragments, powders, or formed articles, or in aqueous solution, so no specific recommendations are given on the test procedures for electrospun materials. So, there is a need for standardization and regulations to ensure the safety, quality, and sustainability of biodegradable electrospun materials. Currently, efforts are being made to establish guidelines and standards for green electrospinning processes, materials, and products. However, future perspectives should also involve the development and implementation of standards and regulations to promote responsible and sustainable electrospinning EoL practices [63].

During the selection of a biodegradable material for an intended application, it may be necessary to refine its rate of degradation to bring it closer to the desired rate. There are several methods to alter the degradation rate of a material, such as using material mixtures, multiple drugs, additives, and nanofillers [63,64]. However, if these characteristics allow a positive and rapid response, one of the limiting conditions regarding the biodegradation of electrospun membranes in packaging could be the high loading of antimicrobial/antifungal agents considered for active food preservation [65]. It is, therefore, requested to thoroughly investigate the responses of electrospun systems towards biodegradation for packaging purposes. On this basis, an analysis of the main sustainable EoL opportunities for electrospun materials has been reported.

### 6.2. Biodegradation Assays

The methodologies currently considered to evaluate the biodegradability of biopolymers can be structured according to the place where the plastics will be disposed, namely in soil, compost, or aquatic systems. However, if the analyses are limited to soil/compost degradation or to decomposition due to enzyme action, different research results on electrospun food packaging materials are available [66,67].

A disintegration test can be conducted under simulated industrial composting conditions at 58 °C to study the compostability of electrospun polymeric materials. According to the ISO 20200 standard [68], a qualitative evaluation of the physical disintegration of fiber mats as a function of the composting time can be performed by taking photographs and SEM measurements. The disintegration degree can also be analyzed in terms of mass loss as a function of disintegration time. For instance, Arrieta et al. reported about the disintegrability of a series of electrospun PLA-PHB mat formulations under composting conditions. Full disintegration in less than 2 months was confirmed for the electrospun materials by the addition of a high content (>15%) of OLA plasticizer [12]. The addition of cellulose nanocrystals (CNCs) [69], chitosan, and catechin [11,70] was also tested, evidencing that all electrospun bionanocomposites were fully disintegrated under composting conditions. Leonés et al. (2021) studied the effects of CNC, hydroxyapatite, and silver nanoparticle incorporation into different PLA-based nanocomposite electrospun fibers (1%wt. with respect to the PLA). All organic and inorganic reinforced systems were disintegrated under composting conditions after 35 days [71].

Triggered degradation due to common environmental factors, such as UV light and soil burial, is, indeed, another adopted methodology suitable for assessing the biodegradation of electrospun materials. The biodegradability of mats in soil varies with their composition (organic/inorganic matters, oxygen/carbon dioxide, and water content), temperature, and pH. Therefore, degradation rate and range can be modified in different regions and seasons. Commonly, mineralization under aerobic conditions by microorganisms produces CO_2_, H_2_O, and biomass, and the quantification of CO_2_ evolution is required to determine the degree of biodegradation. Some studies have shown a faster degradation process in soil for electrospun mats than compacted films. As an example, the influences of UV radiation, water, and soil microbiota on PLA/natural rubber nonwoven fibers were studied, with the results indicating that the addition of natural rubber to the PLA matrix accelerated the degradation process in the soil by increasing the content of the amorphous phase in the composites and the bioavailability of natural rubber, while photodegradation was slowed down by the presence of NR [72]. Blending with PHB was also found to be a possibility for tuning biodegradation. In the paper of Olkhov et al. (2023), the oxidative and biological degradation of PLA/PHB electrospun mats with a PLA content from 30 to 90 wt.% in a liquid inoculated medium was assessed, by confirming that a tuned compositions in terms of degradation kinetics can be used to realize biopackaging [73]. The incorporation of active substance, nanoparticles, and plasticizers, as well as the effect of temperature, favors this process [74,75,76]. Nevertheless, the available literature mostly considers specific degradation in soil as an EoL option for agriculture applications (i.e., mulching films), so limited information is available on mats used for packaging purposes.

The biodegradability of biopolymers also varies in accordance with the accessibility of the structure to moisture and enzyme diffusion and the capacity of the microbes in the environment to assimilate the final monomers. The ease of access to enzymes and water depends on the hydrophobicity, crystallinity, and steric effects of the side groups in the polymer backbone, since enzymes approach the fiber surface and initiate the hydrolysis of the polymer [77]. Maintaining tension during enzyme exposure facilitates improving the polymer chain alignment to the fiber axis, tensile strength, and crystallinity after collection, with a subsequent reduction in degradation rate [78]. The biodegradation of polymeric materials also depends on characteristics such as molecular composition, the presence of functional groups, intermolecular interactions, morphology, configuration, surface structure, and molecular weight [79]. All these factors and environmental conditions play key roles in tuning the biodegradation of electrospun products.

### 6.3. Influence of Factors in Biodegradation

The biodegradation process of electrospun mats is influenced by the physical and chemical characteristics of soil conditions [80]. Furthermore, the presence of antimicrobial/antifungal compounds, fillers, plasticizers, and reinforcements sometimes affects the degradation and disintegration kinetics of polymeric matrixes [80]. The composition and variation of microbial communities in the soil also influence the ecosystem and, consequently, biodegradation. Moreover, the ecological and physical–chemical factors change in the retaliation of soil and in different seasons. It is essential to know how these parameters influence polymeric biodegradation to understand their relative impacts [81].

Disintegration tests in composting conditions carried out according to the ISO 20200:2023 normative involve the use of commercial compost with certain amounts of rabbit food, sawdust, starch, urea, and oil. These tests can be performed in mesophilic (25 °C) or thermophilic (58 °C) conditions, which allow for the growth of microorganisms needed for disintegration [68].

As highlighted in the literature, not all biodegradable polymers are compostable, since compostability involves biodegradation by biological activities at a rate consistent with other known compostable materials, leaving residues that are not visibly distinguishable or toxic [82,83]. Therefore, the determination of compostability kinetics involves the following three phases: disintegration, biodegradation, and ecotoxicity. The biodegradability and compostability of active biodegradable packaging materials can be influenced by the presence of active compounds or polymer blends and cannot be assumed as such.

By using electrospinning, nanofibers with a high surface-to-volume ratio porosity are produced. Surface wettability and porosity play key roles in the degradation pattern of nanofibers, not only in soil. This characteristic is particularly important if the electrospun mats are applied as food packaging materials. Tampau et al. (2020) evaluated the biodegradation and disintegration (under thermophilic composting conditions for 45 and 84 days) of thermoplastic starch multilayers containing polycaprolactone nanofibers loaded with carvacrol. The disintegration of multilayers was similar, reaching values of 75–80% after 84 days. Biodegradation, assessed by CO_2_ measurements, revealed that all the carvacrol-free films completely biodegraded after 25 composting days. Furthermore, carvacrol notably affected the compost inoculum activity and limited the biodegradability of the polymeric active multilayers to a maximum value of around 85% after 45 days [83].

**Figure 3:**
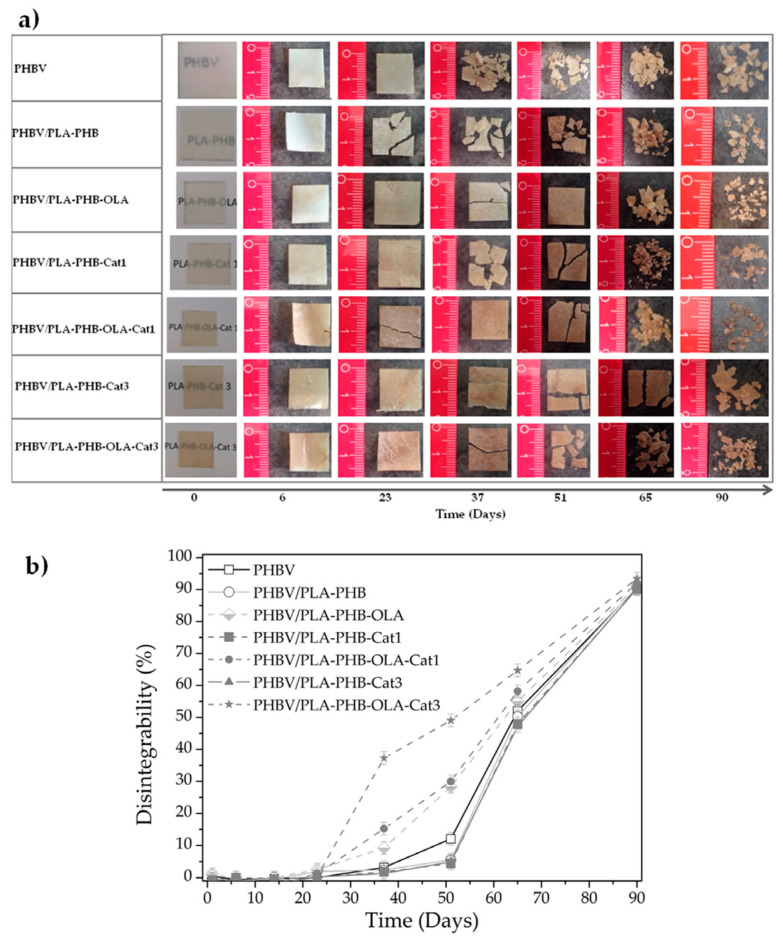
Disintegration of bilayer films at different incubation times under composting conditions: (**a**) visual appearance and (**b**) disintegration kinetics. From Arrieta et al. [11] used under a Creative Commons CC—BY license.

Arrieta et al. (2019) developed bilayer antioxidant systems based on polymer blends loaded with OLA and catechin and evaluated their end of life through disintegration tests under composting conditions at a laboratory-scale level [11]. The outer layer was composed of a compression-molded PHBV material, while the inner layer was composed of electrospun fibers of PLA/PHB blends. OLA was used as a plasticizer and catechin at 1 and 3%wt was used as an antioxidant compound. Figure 3 shows the visual observation and degradation kinetics of the bilayer materials. The biodegradable behavior was dominated by the PHBV layer, since it was thicker than the electrospun layer. After 23 days, the bilayer systems started to become breakable due to the start of the hydrolysis process in the electrospun materials, which continued in the PHBV matrix. Thus, the electrospun layer somewhat sped up the disintegration process. The presence of catechin slightly delayed the disintegration process, while OLA sped it up due to its plasticization effect. Furthermore, all formulations were totally disintegrated under composting conditions in less than three months.

## 7. Active Properties and Food Packaging Applications of PLA- and PHA-Biopolyester-Based Electrospun Materials

Biodegradable electrospun packaging materials based on PLA or PHA have shown potential benefits for the future of the food industry. These materials have been functionalized by the addition of antioxidant and antimicrobial compounds. Also, the development of core–shell structures has promoted a lower volatility and the release of encapsulated compounds [25]. In this context, the selection of polymers for the structures of the core and shell, as well as the amount of active compound incorporated into the core structure, is essential to ensure the sustained release of the compound. Patiño Vidal et. al. developed two different antimicrobial core–shell matrixes, PLA_shell_-PVOH_core_ and PLA_shell_-PLA_core_ loaded with ethyl lauroyl arginate (LAE), into the core structure and studied the release of the active compound towards food simulants. A better affinity between the polymers of the PLA_shell_-PLA_core_ matrix favored a higher incorporation of LAE (15 wt%.) compared to the PLA_shell_-PVOH_core_ matrix with 2.5 wt%. of LAE. Furthermore, the PLA_shell_-PLA_core_ matrix significantly decreased the release of LAE [21,38].

The antioxidant and antimicrobial properties of these electrospun materials have been widely assessed through different methods, but their effectiveness in real conditions has been scarcely evaluated. This section reports both in vitro and in vivo studies of active electrospun materials with food packaging purposes.

### 7.1. Assessment of Antimicrobial and Antioxidant Properties Under In Vitro Properties

Bacterial growth is a major problem for the food industry. Therefore, the application of antibacterial materials able to inhibit the growth of Gram-positive and Gram-negative bacteria is one of the most used strategies to protect food products. Table 2 shows the recent developments regarding PLA and PHA electrospun materials loaded with antimicrobial and antioxidant compounds.

PLA electrospun materials containing synthetic compounds [84], essential oils, and their derivates [85] and natural extracts [86] have been developed. PHBV electrospun mats loaded with essential oils and natural extracts have also been researched [46,87]. Polymers containing antibacterial compounds have been electrospun and converted into uniaxial nanofibers, and their effectiveness against *Staphylococcus aureus* and *Listeria innocua* as Gram-positive and *Escherichia coli*, *Salmonella typhimurium*, and *Salmonella enterica* as Gram-negative bacteria has been tested through direct contact assays such as dynamic contact and disk diffusion. Dynamic contact allows for evaluating the antibacterial effectivity of electrospun nanofibers under microbial growth conditions (37 °C and 150 rpm). In contrast, the disk diffusion method allows for validating the antibacterial efficacy of biodegradable electrospun films through the measurement of the microbial inhibition diameter (halo). Also, international normatives, such as the Japanese Industrial Standard (JIS) Z2801 [88] and ASTM E2149-13-1 [89], have been used to evaluate the antibacterial activity of these materials. In consequence, the results of the antibacterial effects of PLA- and PHA-based electrospun materials have been expressed as logarithmic reductions in bacterial concentration, inhibition percentages of microbial growth, and the microbial inhibition zone. Liu et al. (2018) developed PLA fibers loaded with different ratios of tea polyphenols, and the electrospun materials with the highest concentration of active compounds were able to inhibit the growth of *E. coli* and *S. aureus* by 92% and 95%, respectively [90]. A blend of PLA and polycaprolactone (PCL) with the incorporation of thymol allowed for obtaining PLA/PCL fibers able to generate bacterial inhibition halos of *E. coli* and *S. aureus* between 4 and 9 mm [91]. Likewise, the strong antibacterial effect of PLA fibers loaded with *Allium ursinum* L. extract was demonstrated by the inhibition of the growth of *S. aureus* and *E. coli* by 27% and 73%, respectively [86]. Regarding antibacterial PHBV electrospun materials, the research group of Figueroa-Lopez et al. incorporated cyclodextrin inclusion complexes of OEO, rosemary extract (RE), and green tea extract (GTE) and tested their effectiveness against *S. aureus* and *E. coli*. The research group obtained satisfactory results with logarithmic reductions between 1.9 and 3.6 for *S. aureus* and between 1.2 and 3.5 for *E. coli* [46,87].

The development of coaxial fibers and multilayer materials containing antibacterial electrospun layers of PLA and PHBV has been another approach. Coaxial fibers formed by a shell of PLA and a core of PLA loaded with LAE have expressed a strong antibacterial effect (log reduction higher than seven) against *L. innocua*, *S. aureus*, *E. coli*, and *S. typhimurium* [38]. Regarding biodegradable multilayer materials, electrospun antibacterial layers of PLA or PHBV have been incorporated as intermediate structures. Recently, a study developed a trilayer packaging material composed of an extruded PLA layer, an electrospun layer based on core–shell PLA/PLA fibers loaded with LAE, and a chitosan casted layer. The antibacterial effectiveness of this trilayer material was tested in liquid and solid medium, and both assays validated the strong antibacterial effect of the trilayer material against *L. innocua* and *S. enterica* [13]. Another study deposited a PHBV electrospun layer loaded with oregano essential oil and zinc oxide nanoparticles onto YPACK210 extruded films. Furthermore, the electrospun layer was coated with a CNC layer. The multilayer material promoted a logarithmic reduction of approx. five in *E. coli* and *S. aureus* for 15 days [17].

On the other hand, oxidation is another deterioration phenomenon of several foods. To solve this drawback, essential oils, natural extracts, and synthetic compounds have been incorporated into PLA, PHB, PHBV, and PLA/PCL uniaxial fibers [11,91,92,93]. The antioxidant activity of these materials has been largely measured through the DPPH test, which is based on the free radical scavenging activities of DPPH (the bleaching rate of the stable radical 2,2-diphenyl-1-picrylhydrazyl). For instance, OEO, RE, and GTE were encapsulated into PHBV electrospun fibers, and these materials produced a DPPH inhibition rate between 22 and 43% [87]. Likewise, electrospun PLA/PCL fibers loaded with thymol were able to inhibit 50% of DPPH radicals [91]. Curcumin and quercetin have also been incorporated into PHB fibers, and these electrospun materials inhibited between 16 and 34% of DPPH radicals. On the other hand, ABTS and CUPRAC assays have also been used to evaluate the antioxidant activity of materials. ABTS is a method based on the free radical scavenging activities of ABTS (the inhibition of the cationic radical 2,2-azinobis(3-ethylbenzothiazoline-6-sulfonate)), while CUPRAC is a method based on the reduction of cupric (Cu^+2^) to cuprous (Cu^+^) [8,94]. The DPPH, ABTS, and CUPRAC methods were recently used to evaluate the antioxidant activity of PLA uniaxial fibers loaded with lavender essential oil. The results were expressed as the IC_50_ for DPPH and ABTS and as A_0.5_ for CUPRAC, and the values obtained showed the strong antioxidant activity of electrospun materials.

Biodegradable multilayer materials with antioxidant properties have also been developed. Electrospun layers have acted as antioxidant intermediate layers in multilayer structures. Figueroa-Lopez et al. (2020) developed an antioxidant multilayer material incorporating an intermediate electrospun layer of PHBV loaded with OEO and zinc oxide nanoparticles between layers of CNC and YPACK210 films. The multilayer material was able to inhibit DPPH radicals for 15 days [17]. Likewise, bilayer materials composed of a PHBV compression molded film and electrospun PLA/PHB fibers loaded with catechin showed a high antioxidant activity for 20 days [11].

As has been described in this section, PLA- and PHA-biopolyester-based electrospun materials with antioxidant and antimicrobial properties are highly suitable for active food packaging. However, their effectiveness must be evaluated on real food matrixes. The next section summarizes some applications of biodegradable electrospun materials in foods.

### 7.2. Food Packaging Applications

As Table 2 shows, recent developments regarding biodegradable active electrospun materials have evaluated their antimicrobial and antioxidant properties on fruits, bread, fish, and meat. PLA and essential oils have been processed to obtain active uniaxial nanofibers. Strawberry has been the most studied fruit, since it is considered to be a highly perishable food [95]. For instance, strawberries were packaged without direct contact with PLA nanofibers loaded with thyme essential oil and stored at 25 °C for 5 days. The results evidenced that electrospun material delayed the weight loss and firmness of the strawberries. Furthermore, mold growth in the fruits was inhibited during storage time [42]. Similarly, Rusková et al. (2023) also packaged strawberries without direct contact with PLA/PHB nanofibers containing OEO and lemongrass EO and stored at 4 and 85% relative humidity for 21 days. The ripening process and the mold growth in the fruits slowed down, and the strawberries showed greater acceptance and quality characteristics [85].

On the other hand, the effectiveness of active mats in the preservation of fish and meat products has been also evaluated. The shelf lives of salamander fillets, pork, and beef were extended by the action of cyclodextrin inclusion complexes of octyl gallate, essential oils, and α-tocopherol. Salamander fillets were wrapped with PLA nanofibers loaded with cyclodextrin inclusion complexes of octyl gallate and stored at 4 °C for 15 days. The antibacterial electrospun film was able to inhibit the growth of *E. coli* by a 72% [96]. The shelf life of pork was effectively prolonged by using PLA nanofibers loaded with cyclodextrin inclusion complexes of cinnamon essential oil. The total viable microbial counts of pork packaged with the electrospun mat and stored at 25 °C for 8 days were decreased by 3 log CFU/g sample [97]. For beef, the incorporation of a cyclodextrin inclusion complex of α-tocopherol into PLA nanofibers decreased 50% of the lipid oxidation of the samples during 21 days of storage [92].

The shelf life of bakery products has been also prolonged. The study conducted by Altan et al. (2018) developed PLA fibers loaded with carvacrol able to inhibit the growth of molds and yeasts in whole wheat bread samples. Samples of bread were kept without direct contact with the antifungal electrospun film and stored at 25 °C for 7 days. The results evidenced that the mold and yeast growth was inhibited up to 100% during storage [98].

**Table 2:** Recent studies on antioxidant, antimicrobial, and food packaging applications of PLA and PHA electrospun materials.

Antimicrobial Properties				
Electrospun Structure	Active Compound	Methodology	Main Findings	Reference
PHBV uniaxial fibers	Cyclodextrin inclusion complexes of OEO	Japanese Industrial Standard (JIS) Z2801	Log reduction *S. aureus:* 3–3.6 *E. coli:* 2.5–3.5	[46]
PLA uniaxial fibers	*Allium ursinum* L. extract	Direct contact	Microbial growth inhibition *S. aureus:* 27% *E. coli:* 73%	[86]
PHBV uniaxial fibers	OEO, RE and GTE	Japanese Industrial Standard (JIS) Z2801	Log reduction *S. aureus:* 1.9–3.2 *E. coli:* 1.2–2.9	[87]
PLA uniaxial fibers	Tea polyphenols	Dynamic contact	Microbial growth inhibition *S. aureus:* 80–95% *E. coli:* 65–90%	[90]
PLA/PCL uniaxial fibers	Thymol	Disk diffusion	Microbial inhibition *S. aureus:* 4.6–6.2 mm *E. coli:* 4.1–9.1 mm	[91]
PLA-PLA coaxial fibers	LAE	Normative ASTM E2149-13-1.	Log reduction *L. innocua:* 7.3 *S. aureus:* 7.4 *E. coli:* 8.0 *S. typhimurium:* 7.8	[38]
PLA_extruded_-PLA/PLA-LAE_electrospun_-Chitosan_casted_ Multilayer material	LAE	Dynamic contact Disk diffusion	Total inhibition of microbial growth of *S. enterica* and *L. innocua* for 15 days. Microbial inhibition *S. enterica:* 27 mm *L. innocua:* 30 mm	[13]
YPACK210_extruded_-PHBV_electrospun_-CNC_coating_-YPACK210_extruded_ Multilayer material	OEO and zinc oxide nanoparticles	Japanese Industrial Standard (JIS) Z2801	Log reduction ≈ 5 to *S. aureus* and *E. coli* for 15 days.	[17]
**Antioxidant Properties**				
**Electrospun Structure**	**Active Compound**	**Methodology**	**Main Findings**	**Reference**
PLA/PCL uniaxial fibers	Thymol	DPPH	DPPH inhibition: 20–55%	[91]
PLA uniaxial fibers	α-Tocopherol and cyclodextrin inclusion complexes of α-Tocopherol	DPPH	DPPH inhibition: 4–97%	[92]
PLA uniaxial fibers	Clove and argan oils	DPPH	DPPH inhibition of 80%	[93]
PHBV uniaxial fibers	OEO, RE and GTE	DPPH	DPPH inhibition: 22–43%	[87]
PLA uniaxial fibers	Lavender essential oil	DPPH, ABTS and CUPRAC	DPPH: IC_50_ 29–47 * ABTS: IC_50_ 14–35 * CUPRAC: A_0.5_ 22–40 *	[99]
PHB uniaxial fibers	Curcumin and quercetin	DPPH	DPPH inhibition: 16–34%	[100]
PHBV_molded_-PLA/PHB_electrospun_ Multilayer material	Catechin	DPPH	Strong DPPH inhibition for 20 days	[11]
YPACK210_extruded_-PHBV_electrospun_- CNC_coating_-YPACK210_extruded_ Multilayer material	OEO and zinc oxide nanoparticles	DPPH	DPPH inhibition: 6–12%	[17]
**Food Packaging Applications**				
**Polymer**	**Active Compound**	**Fiber Architecture**	**Food Application**	**Reference**
PLA/PHB	Oregano and lemongrass EOs	Uniaxial	Strawberry	[85]
PLA	Pectin + thymol	Uniaxial	Citrus Reticulata Blanco	[101]
PLA	Thyme EO	Uniaxial	Strawberry	[42]
PLA	LAE	Uniaxial	Strawberry	[102]
PLA	β-cyclodextrin inclusion complexes of octyl gallate	Uniaxial	Salamander	[96]
PLA	Carvacrol	Uniaxial	Bread	[98]
PLA	Cyclodextrin inclusion complex of α-tocopherol	Uniaxial	Beef	[92]
PLA	β-cyclodextrin inclusion complexes of cinnamon EO	Uniaxial	Pork	[97]

OEO: oregano essential oil; RE: rosemary extract; GTE: green tea extract; LAE: ethyl lauroyl arginate; CNC: cellulose nanocrystals. EOs: essential oils. Results expressed as: * ug/mL.

## 8. Recent Patents for PLA- and PHA-Biopolyester-Based Electrospun Materials for Food Packaging

The accelerated technological readiness level of electrospinning is reflected by an increase in patent requests associated with biodegradable food packaging materials since 2019. According to Table 3, China is the country with the highest number of registered patents. Most of these patents involve the use of uniaxial electrospinning to obtain antimicrobial, antiviral, and intelligent mono- and multilayer films. Coaxial electrospinning has also been used to produce active PLA electrospun films.

The intellectual property of electrospun packaging materials has mainly implicated the use of PLA as a biodegradable polymer, followed by PHB, PHBV, poly(3-hydroxybutyrate-co-valerate) with a molar concentration of 20% hydroxyvalerate (PHBV20), and poly(3-hydroxybutyrate-co-valerate) with a molar concentration of 8% hydroxyvalerate (PHBV8). Inventions have also considered the incorporation of natural extracts, essential oils, and synthetic compounds to afford antimicrobial properties to films. Furthermore, the development of multilayer films (two, three, or four layers) is the best strategy for producing biodegradable electrospun materials with excellent water vapor and gas barrier properties and long-lasting active effects.

Table 3 details the latest patents on biodegradable and electrospun food packaging materials. In this framework, the composition and functionality of electrospun prototypes were mainly addressed. The goal was to show the advancement of electrospinning to encourage its use even more in the food packaging area through prototypes able to be scaled to the industry.

**Table 3:** Recent patents of biodegradable electrospun materials for food packaging.

Title of Patent	Electrospinning Processing	Description	Publication/Applicant
Polylactic acid/citral electrostatic spinning nanofiber membrane as well as preparation method and application thereof	Coaxial	Electrospun film composed by coaxial nanofibers with a shell of PLA and a core of citral able to maintain the quality and freshness of turbot fillets under refrigeration conditions	CN116377653A (2023), Qingdao Agricultural University; Qingdao Naval Food and Nutrition Innovation Institute, Qingdao, China. [103]
Preparation method of antibacterial and antiviral packaging material and packaging material applying antibacterial and antiviral packaging material	Uniaxial	Antibacterial and antiviral electrospun film based on modified PLA nanofibers containing ryegrass extract incorporated on modified mesoporous silica	CN115928318A (2023), South China Agricultural University, Guangzhou Startec Science & Technology Co., Ltd., Guangzhou, China. [104]
Nanometer intelligent indicating film with dual effects of meat preservation and freshness visualization and preparation method of nanometer intelligent indicating film	Uniaxial	Intelligent and antibacterial bilayer film structured by an electrospun layer of PHBV nanofibers containing ferulic acid and a coating layer composed of PVOH, konjac glucomannan, and alizarin, with low permeability to water vapor	CN116446110A (2023), Chengdu University, China [105]
Lauroyl arginine ethyl ester hydrochloride antibacterial nanofiber membrane and ultrahigh pressure combined sterilization method	Uniaxial	Electrospun film composed by antibacterial nanofibers of PLA with LAE able to improve the efficiency of ultrahigh pressure sterilization	CN116326711A (2023), China Agricultural University, China [106]
Nano-composite preservative film with thermosensitivity and preparation method thereof	Uniaxial	Electrospun trilayer film structured by two outer layers of PVOH and PNIPAAm and an intermediate layer of PLA loaded with lemon essential oil, with good thermosensitivity and excellent protection for blackberries stored between 25 °C and 35 °C and a relative humidity of 80%	CN115447230A (2022), Nanjing Forestry University, China [107]
Biodegradable container, method for obtaining same and use thereof for contact, transport, and/or storage of perishable products	Uniaxial	Water vapor and gases barrier and antibacterial multilayer trays composed by four layers: (i) an extruded and structural of PHB and lignocellulose, (ii) an electrospun and barrier to water and gases of EVOH44, (iii) an electrospun of PHBV20 and oregano essential oil, and (iv) an extruded of PHBV8.	US20220195248A1 (2022), NASTEPUR, S.L., Valencia, Spain; USA [108]
Preparation method and application of slow-release multilayer composite fiber membrane	Uniaxial	Multilayer electrospun composite film composed by two outer layers of PLA and an intermediate layer of PLA loaded with tea polyphenols, with long-lasting and fresh-keeping effect on strawberries	CN113265765A (2021), North University of China, China [109]
Nanostructures based on PHBV and ZnO nanoparticles doped with Fe and process for preparing the same	Uniaxial	Antimicrobial bilayer film composed by an extruded PLA layer and an electrospun layer of PHBV loaded with ZnO nanoparticles doped with Fe ions, whose values of specific migration of ZnO and Fe towards food simulants were within allowed limits	RO134634A0 (2020), Institutul Naţional de Cercetare-Dezvoltare Pentru Tehnologii Izotopice şi Moleculare, Rumania [110]
Compositions for controlled release of volatile compounds	Uniaxial	Active electrospun films based on polymer blends of PLA/PEO, EC/PEO, and CA/PEO loaded with cinnamaldehyde, thymol, and carvacrol, with controlled release of volatile compounds	US20190200651 (2019), University of Guelph, USA [111]

PNIPAAm: N-isopropylacrylamide. EVOH44: poly(ethylene-co-vinyl alcohol) with ethylene content of 44 mol%. EC: ethyl cellulose. CA: cellulose acetate.

## 9. Conclusions and Future Remarks

The development of PLA- and PHA-biopolyester-based food packaging materials has been presented as a suitable alternative to address the high accumulation of fossil-based plastic material waste. The combination of the valuable physical–chemical, mechanical, optical, and barrier properties of biodegradable polymers and the use of electrospinning is the most recent and innovative proposal for the packaging industry.

Electrospinning is an efficient, cost-effective, and scalable technology able to produce nanofiber- and microfiber-based films with a high surface-to-volume ratio. Through this technology, electrospun films have been functionalized (by adding active compounds) or reinforced (by incorporating nanoparticles or inorganic substances). Uniaxial, coaxial, emulsion, multiaxial, and needleless electrospinning have allowed for obtaining packaging materials. However, uniaxial and coaxial electrospinning have been exclusively used to develop functionalized and reinforced PLA- and PHA-biopolyester-based electrospun materials. These materials have been developed as a single structure or have been part of multilayer systems as intermediate layers. Furthermore, the addition of active compounds and reinforcements has mainly enhanced their thermal, barrier, surface, and mechanical properties.

The biodegradability of PLA- and PHA-biopolyester-based electrospun materials has been mainly evaluated in soil, compost, and aquatic systems through ASTM and ISO normatives. The biodegradation process of materials is dependent on the polymer type, conditions, and test methods. The physical and chemical characteristic of soil conditions, the presence of antimicrobial compounds, fillers, plasticizers, reinforcements, the composition, and variation of microbial communities in the soil, as well ecological and physical–chemical factors, have affected the degradation and disintegration kinetics of polymeric matrixes.

On the other hand, single and multilayer biodegradable electrospun structures have exhibited antimicrobial and antioxidant properties thanks to the addition of synthetic compounds, essential oils and their derivates, and natural extracts to fibers. Biodegradable electrospun materials have shown a strong antibacterial activity against Gram-negative and Gram-positive bacteria and prolonged antioxidant activity. The positive benefits of these materials have promoted their application for real food. Thus, the shelf lives of fruits, bread, fish, and meat have been satisfactorily extended. Finally, the accelerated technological readiness level of electrospinning is reflected by an increase in patent requests associated with biodegradable food packaging materials. China is the country with the highest number of registered patents. Intellectual property has mainly involved the use of PLA, PHB, PHBV, PHBV20, and PHBV8 as polymers and natural extracts, essential oils, and synthetic substances as antimicrobial compounds. Furthermore, biodegradable electrospun films with multilayer structures have shown excellent water vapor and gas barrier properties and long-lasting active effects.

Through this critical review, new developments regarding biodegradable electrospun materials for food packaging purposes can be proposed as alternatives to fossil-based packaging.
